# Numerical analysis for tangent-hyperbolic micropolar nanofluid flow over an extending layer through a permeable medium

**DOI:** 10.1038/s41598-023-33554-9

**Published:** 2023-08-19

**Authors:** Galal M. Moatimid, Mona A. A. Mohamed, Ahmed A. Gaber, Doaa M. Mostafa

**Affiliations:** 1 Department of Mathematics, Faculty of Education, Ain Shams University, Roxy, Cairo, Egypt; 2 Department of Mathematics, College of Science, Qassim University, P. O. Box 6644, Buraidah, 51452 Saudi Arabia; 3 Department of Mathematics, College of Science and Humanities at Howtat Sudair, Majmaah University, Majmaah, 11952 Saudi Arabia

**Keywords:** Energy science and technology, Engineering, Materials science, Mathematics and computing

## Abstract

The principal purpose of the current investigation is to indicate the behavior of the tangent-hyperbolic micropolar nanofluid border sheet across an extending layer through a permeable medium. The model is influenced by a normal uniform magnetic field. Temperature and nanoparticle mass transmission is considered. Ohmic dissipation, heat resource, thermal radiation, and chemical impacts are also included. The results of the current work have applicable importance regarding boundary layers and stretching sheet issues like rotating metals, rubber sheets, glass fibers, and extruding polymer sheets. The innovation of the current work arises from merging the tangent-hyperbolic and micropolar fluids with nanoparticle dispersal which adds a new trend to those applications. Applying appropriate similarity transformations, the fundamental partial differential equations concerning speed, microrotation, heat, and nanoparticle concentration distributions are converted into ordinary differential equations, depending on several non-dimensional physical parameters. The fundamental equations are analyzed by using the Rung-Kutta with the Shooting technique, where the findings are represented in graphic and tabular forms. It is noticed that heat transmission improves through most parameters that appear in this work, except for the Prandtl number and the stretching parameter which play opposite dual roles in tin heat diffusion. Such an outcome can be useful in many applications that require simultaneous improvement of heat within the flow. A comparison of some values of friction with previous scientific studies is developed to validate the current mathematical model.

## Introduction

Because of the continuous advancements in manufacturing, non-Newtonian fluids have attracted academic attention during the last decades. Coal–oil paints, intelligent coatings and formulations, cosmetics, and physiological liquids are only a few examples of such fluids. Non-Newtonian fluids do not have a specific fundamental correlation involving strain rate and stress. This is because of the wide range of properties of these liquids in the environment. These fluids have much more challenging mathematical problems than viscous fluids due to dangerous higher-order nonlinear differential equations. Although numerical approaches are normally essential to solve the mathematical combinations that emerge in the non-Newtonian prototypes, analytically restricted approaches have been found in a few instances. Exact and numerical outcomes provide valuable support for experimental investigations. A tangent hyperbolic fluid surrounding a sphere subjected to a convective boundary condition and a Biot number was the subject of discussion in regard to Brownian motion and thermophoresis consequences^1^. There hasn’t been much research done on concentration boundary conditions involving a wall normal flow of zero nanoparticles. Investigations were done into how the mixed convection tangent hyperbolic flow was affected by radiation absorption and activation energy^2^. When the radiation absorption and activation energy parameters were raised, it was discovered that the velocity improved. The movement and temperature transmission of an incompressible tangent hyperbolic non-Newtonian flow through a normal porous cone and a magnetic strength was analyzed in a nonlinear non-isothermal steady-state border sheet^3^. In the existence of thermal and hydrodynamic slip, the thermostatic sphere's nonlinear continuous border sheet flow and temperature exchange of an incompressible tangent hyperbolic non-Newtonian liquid were studied^4^. A tangent hyperbolic nanofluid flowing cylinder with Brownian movement and thermophoresis influences in an unstable MHD free convection flow was explored^5^. The motivation of this study was to keep coming up with numerical formulations for a time-responsive incompressible tangent hyperbolic fluid as well as nanoparticles in the context of a moving cylinder. The movement of a tangent hyperbolic liquid along a flow of an expanding layer was studied^6^. The use of nonlinear radiation was used to enhance heat transfer properties. The energy was used to characterize additional aspects of mass transfer. By incorporating the relevant laws, the situation was modeled from the perspective of boundary layer equations. The impact of changing thermal conductivity on the MHD tangent hyperbolic liquid in the existence of nanoparticles through a stretched surface was investigated^7^. The combined stimulation of slip and convection circumstances with heat generation, viscous dissipation, and Joule heating was inspected for heat and mass transmission processes. Recent work has used an appropriate rheological model to investigate the stagnation point movement and thermal properties of a tangent hyperbolic liquid across a normal border^8^. A tangent hyperbolic liquid movement prototype was used to simulate the physical circumstance. A new approach for translating the important formulations of a double-diffusive MHD hyperbolic tangent liquid prototype hooked on a set of nonlinear fundamental formulae was proposed, using the Lie group analysis procedure^9^. In accordance with the previous aspects, the current work is conducted through the tangent hyperbolic fluid flow.

Because of the numerous applications of micropolar fluid motion in plasmas, furnaces design, and nuclear power plants over the past several decades, have attracted a lot of attention. Micropolar fluids including an asymmetric stress tensor that can really continue rotating according to the conservation laws of the reflecting non-Newtonian fluid description were a subclass of micropolar fluids. Fundamentally, such substances were defined as fluids made up of colloidal matter with random orientations in a viscous medium. The movement of infected animals, liquid crystals, suspending treatments, and heterogeneous liquids can all be better understood using this fluid approach. The unsteady flow of a micropolar fluid over a curving stretched surface was taken into consideration with regard to heat and mass transfer^10^. It looked at the consequences of thermophoresis and Brownian motion. On the curved surface, the impacts of suction/injection situations were also discussed. Thermodynamic limitations have also been thoroughly examined^11^. In the conclusion of his book on the hypothesis and presentations of micropolar liquids, it was outlined a number of interesting characteristics. Using a vertically nonlinear Riga stretched sheet, a comparative investigation of the flow of micropolar Casson nanofluid was examined^12^. Under thermophoresis and Brownian movements, the influences of temperature and velocity slip were taken into consideration. The fluid velocity distribution curves were discovered to exhibit rising behavior as a result of micropolar parameter changes. The hydromagnetic radiative peristaltic blood phenomena of a micropolar fluid along a channel using the Adomian decomposition method was explored^13^. The effects of different settings were visually shown. Additionally, the micropolar liquid model seems to be more appropriate for biofluids like blood. Peristalsis has received a great deal of interest in the field of fluid mechanics in recent years due to its importance in physiological technologies and advanced implementations. Therefore, a model of a micropolar-Casson fluid following the peristaltic processes involving radiant heat in a symmetrical channel was created, using the lubricating approximation theory^14^. Micropolar fluids included a wide variety of polymeric formulations, lubrication liquids, colloidal extensions, and complexes. Significant applications such as viscous dissipation, heat generation, and slipping situations have an impact on the MHD micropolar liquid flow and temperature transmission over a stretched surface examined^15^. Flow and heat transfer of micropolar fluids via an extended layer in a Darcy permeable material was studied^16^. The Rolex boundary conditions, and the isothermal wall were mostly used to analyze the heat exchange event.

Permeable media were solid matrices with voids (pores) that frequently overflow through water. It was understood that rigid and open-cell porous media were saturated when all of the pores were filled with fluid, allowing the fluid to pass through the voids. Lately, the method of employing nanofluid and permeable media has drawn a lot of interest and has stimulated a lot of studies in this discipline. The surface area of interaction between liquid and solid surfaces was increased by porous media, and the effective heat conductivity is increased by nanoparticles dispersed in a nanofluid. It followed that mixing porous media with nanofluid can greatly boost the effectiveness of conventional thermal systems. An in-depth discussion of the hybrid nanofluid of natural convection was introduced^17^. They tried to determine which nanoparticle model, mono or hybrid, produced a better fluid flow behavior. An evaluation of the nanofluid movement in permeable media was done^18^. It looked at some findings of an MHD liquid in permeable media. Several scientists worked to enhance temperature transmission in free, forced, and mixed convection using nanofluids in porous media^19^. The convection of nanofluids in thermally unstable permeable media embedded in microchannels was studied^20^. For both the liquid and solid stages, temperature distributions in two dimensions were determined. A mixture of permeable media and nanofluids was employed to increase the temperature transmission across a normal cylinder that produced a high heat flux^21^. This process indicated that the electrical apparatus functions intended within the parameters set by the manufacturer. Yirga and Shankar^22^ investigated Soret interactions, viscous dissipation, chemical processes, and convective thermophysical properties in a nanofluid movement across permeable media generated by an extending layer according to a magnetic strength. The mathematical statements were converted to ordinary differential equations using similarity transformations, and the Keller box approach was then used to numerically solve them. The impact of an inclined magnetic field on the Casson nanofluid across an extended layer enclosed in a saturating permeable matrix in the existence of heat transfer and a non-uniform convectively heated layer was investigated^23^. The numerical Runge–Kutta solution using a shooting strategy was used to reach the main conclusions. The effect of thermal radiation dissipation on nanofluids in an unsteady MHD occupied by a permeable medium only along the upstanding conduit was examined^24^.

The high-order differential equations of boundary value problems (BVPs) are one of the most important models that describe many scientific phenomena in diverse areas of physics and engineering. Many researchers have been interested in discovering and developing many mathematical methods for solving these equations^25^. One of these methods is the shooting method, which was developed to solve high-order BVPs easily by dividing the high-order differential equivalence into a structure of first-order differential equalities. The shooting approach can be simply used for nonlinear second order BVP in general. This is the benefit of utilizing the shooting technique over the finite difference method, which requires the solution of finite difference equations^26^. Therefore, this method proved to be effective for solving this type of equation. In recent decades, many researchers have utilized the shooting method to solve BVPs equations. Seddeek et al.^27^, for example, analyzed the flow of magneto micropolar liquid under the effect of radiation. Aurangzaiba et al.^28^ also solved a model of micropolar fluid including temperature transmission. Ibrahim et al.^29^ scrutinized the movement of a viscoelastic nanofluid. Further, Preeti and Ojjela^30^ studied MHD boundary layer flow for a hybrid nanofluid.

The focus of the current work is on understanding how a nanoparticle-containing fluid flows through an extending horizontal sheet at the bottom of a micropolar non-Newtonian fluid. The objective of the current work is to illustrate a coupled-model fluid consisting of the tangent-hyperbolic and micropolar types in addition to dissolved nanoparticles. This model is extremely useful in technologies and production operations, such as rotating metal, making rubber sheets, making glass fibers, producing wire, extruding polymer sheets, production polymers, etc. The discussed problem is thought to give a new orientation to these applications by adding new categories of practical fluids. Within those situations, the rate of cooling and the procedure of extending determine the final desired qualities of the product. As a result, temperature transfer should be taken into consideration, in addition to nanoparticle volume fraction distribution across the tangent-hyperbolic micropolar fluid. Additionally, this work investigated ohmic dissipation, temperature production, magnetic strength, and chemical processes. Ohmic heating dissipation has many applications such as; lightning, melting, recognition of starch gelatinization, cracking, vaporization, dryness, extraction and fermentation, so many of references and the work in hand interest to illustrate its involvement with liquid flows. Situations involving speed, heat, and nanoparticle sliding are designated for the surface. The new findings of the current study are compared with those established in the literature.

The current study attempts to answer the following questions:

- How does the speed of a tangent-hyperbolic micropolar nanofluid respond in the extending layer?
- How are the distributions of temperature and nanoparticles throughout the treated flow organized?
- What are the frequent relationships between the distributions of nanoparticles and microrotation velocity, velocity, and heat?
- What effects do the relevant parameters have on the aforementioned distributions, and what uses are there for them?

The rest of the manuscript is planned as follows to crystallize the demonstration: "Prototype formulation" Section explains the issue approach. The regulating equations of motion, the physical quantities of interest, and the suitable similarity transformations are included in this section as subsections "Description of the boundary-value problem", "Important physical quantities", and "Convenient conversions of relationship", correspondingly. "Mathematical technique" Section is dedicated to introducing the methodology of the shooting method as the numerical utilized technique. "Findings and interpretation" Section presents the findings and discussions. Finally, in "Concluding remarks" Section, the significant findings are summarized as concluding observations.

## Prototype formulation

**Figure 1:**
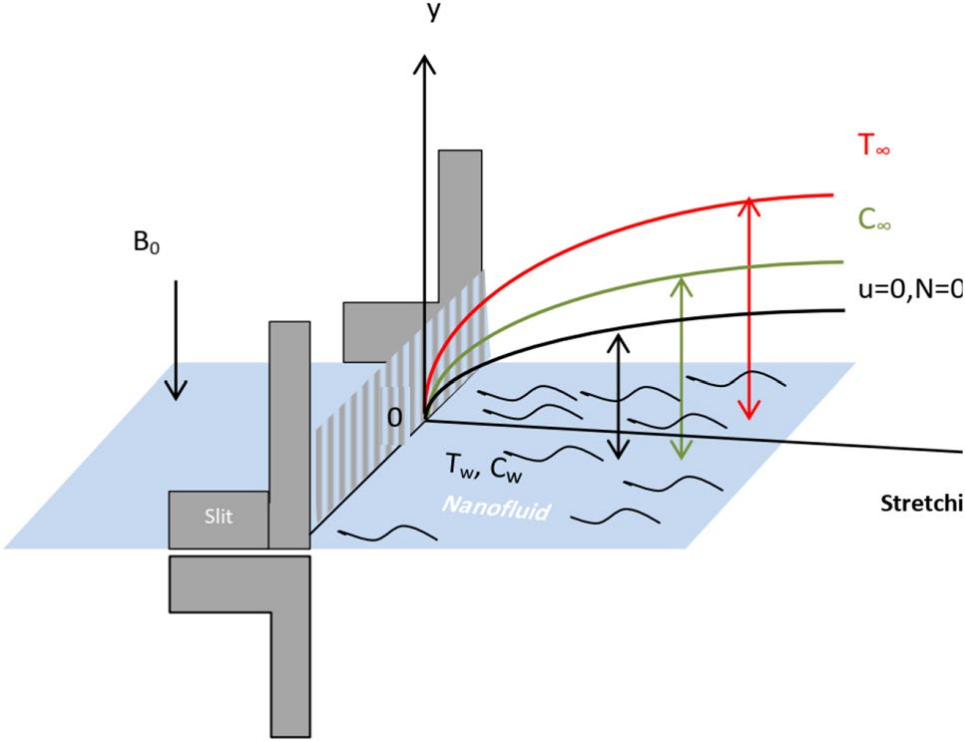
Physical model of the problem.

The present model illustrates a non-Newtonian laminar hydrodynamic two-dimensional nanofluid movement in the neighborhood of a broadening surface, and obeys the tangent hyperbolic prototype^31^ and^32^. The novelty of this work lies in identifying and modeling the thermal and volumetric nanoparticle distributions of the tangent hyperbolic micro rotating liquid across an extending layer. The Cartesian coordinate model is employed, where the expanding border is horizontally aligned along $x -$axis that has a spreading speed $U_{w} = cx$, and the $y -$axis is vertically directed along with the plate as shown in the sketching model Fig. 1. Therefore, the stretching surface is located at $y = 0$, which stretches along with the $x -$path with a steadily stretched parameter, see^31^ and^33^. The flow is supposed to be restricted to the boundary layer region $y > 0$, which is adjacent to the linear spreading border through a permeable medium with permeability $K$. The sheet is maintained at a fixed heat and nanoparticles concentration $T_{w}$ and $C_{w}$, correspondingly. Meanwhile, as $y$ goes to endlessness, the ambient amounts of heat and concentration approaches $T_{\infty }$ and $C_{\infty }$, correspondingly. In this configuration, the flow exhibits the velocity, heat, and mass slip at the surface wall. Along with the normal axis to the stretching surface, a uniform magnetic strength of intensity $B_{0}$ is considered. For the purpose of simplicity, the influence of electric strength can be overlooked. The non-existence of the induced magnetic intensity is produced by the hypothesis of a small Reynolds numeral^31^ and^32^. Because of the presence of the Lorenz force, the fluid is magnetized. One of the most important applications of our model is the flowing fluid over the stretching sheet inside the parabolic trough solar collector which is used in solar cell systems like solar water pumps, solar aircraft wings…etc. Jamshed et al.^34^ and Jamshed et al.^35^ observed that the application of nanofluids and hybrid nanofluids improved thermal transfer, and hence improved the efficiency of the solar cell. The relationship between our discussed model and this real application is that the current flow is studied on a stretching sheet utilizing nanoparticles such as Jamshed. Moreover, the assumed fluid is tangent hyperbolic and micro rotating one under effects of the magnetic field, Ohmic dissipation, heat resource, thermal radiation, and chemical reaction.

### Description of the boundary-value problem

The tensor of Cauchy stress $\overline{\tau }$ is used for hyperbolic tangent fluid and is defined by Ullah et al.^36^ as follows:

1 $$ \overline{\tau } = [\mu_{\infty } + (\mu_{\infty } + \mu_{0} )\tanh (\Gamma \overline{\gamma } )^{n} ]\overline{\gamma } $$

since $\overline{\tau }$,$\mu_{\infty }$, $\mu_{0}$, $\Gamma$ and $n$ denote the tensor of additional stress, the endless shear rate viscosity, the zero shear rate viscosity, the time related material amount and power law index number, correspondingly. The stress tensor $\overline{\tau }$ may be formulated as given by Zakir Ullah et al.^36^:

2 $$ \overline{\tau } = \sqrt {\tfrac{1}{2}\sum\nolimits_{i} {\sum\nolimits_{j} {\overline{\gamma }_{ij} \overline{\gamma }_{ij} } } } = \sqrt {\tfrac{1}{2}\prod } , $$

where $$\prod = \frac{1}{2}{\text{trac}}\left( {\nabla V + (\nabla V)^{T} } \right)^{2}$$.

For simplicity, the case $\mu_{\infty } = 0$ is only considered, i.e., the infinite shear rate viscosity is ignored. Furthermore, as the tangent hyperbolic liquid defines the shear weakening occurrences, thus $\Gamma \overline{\gamma } < 1$ is assumed. Taking these abovementioned assumptions into account, Eq. (1) will take the following form:

3 $$ \overline{\tau } = [\mu_{0} (\Gamma \overline{\gamma } )^{n} ]\overline{\gamma } = [\mu_{0} (1 + \Gamma \overline{\gamma } - 1)^{n} ]\overline{\gamma } \cong [\mu_{0} (1 + n(\Gamma \overline{\gamma } - 1))]\overline{\gamma } $$

The governing equations are assumed to judge an incompressible tangent hyperbolic nanofluid in the description of the current model and are reduced as follows:

The preservation of mass and momentum of an incompressible non–Newtonian fluid may be described as follows^37^:

4 $$ \frac{\partial u}{{\partial x}} + \frac{\partial u}{{\partial y}} = 0 $$

and

5 $$ u\frac{\partial u}{{\partial x}} + \upsilon \frac{\partial u}{{\partial y}} = \left( {(1 - n)\nu + \frac{\kappa }{\rho }} \right)\frac{{\partial^{2} u}}{{\partial y^{2} }} + \sqrt 2 vn\Gamma \frac{\partial u}{{\partial y}}\frac{{\partial^{2} u}}{{\partial y^{2} }} - \left( {\sigma \frac{{B_{0}^{2} }}{\rho } + \frac{v}{L}} \right)u + \frac{\kappa }{\rho }\frac{\partial N}{{\partial y}} $$

The microrotation momentum equation is written by Mohamed and Abou-zeid^38^ as follows:

6 $$ u\frac{\partial N}{{\partial x}} + \upsilon \frac{\partial N}{{\partial y}} = \frac{\gamma }{\rho j}\frac{{\partial^{2} N}}{{\partial y^{2} }} + \frac{\kappa }{\rho j}\left( {2N - \frac{\partial u}{{\partial y}}} \right) $$

The energy and the nanoparticle volume fraction equations are specified by Rehman et al.^39^ as:

7 $$ u\frac{\partial T}{{\partial x}} + \upsilon \frac{\partial T}{{\partial y}} = \frac{\alpha }{{(\rho c)_{f} }}\frac{{\partial^{2} T}}{{\partial y^{2} }} + \frac{{(\rho c)_{p} }}{{(\rho c)_{f} }}\left( {D_{B} \left( {\frac{\partial T}{{\partial y}}\frac{\partial C}{{\partial y}}} \right) + \frac{{D_{T} }}{{T_{\infty } }}\left( {\left( {\frac{\partial T}{{\partial x}}} \right)^{2} + \left( {\frac{\partial T}{{\partial y}}} \right)^{2} } \right)} \right) + \sigma \frac{{B_{0}^{2} }}{{(\rho c)_{f} }}u^{2} - \frac{1}{{(\rho c)_{f} }}\frac{{\partial q_{r} }}{\partial y} + \frac{{Q_{0} }}{{(\rho c)_{f} }}(T - T_{\infty } ) $$

and

8 $$ u\frac{\partial C}{{\partial x}} + \upsilon \frac{\partial C}{{\partial y}} = D_{B} \left( {\frac{{\partial^{2} C}}{{\partial y^{2} }}} \right) + \frac{{D_{T} }}{{T_{\infty } }}\left( {\frac{{\partial^{2} T}}{{\partial y^{2} }}} \right) - R_{1} (C - C_{\infty } ). $$

Rosseland computation^40^ is employed to represent the radiative temperature flux as follows:

9 $$ q_{r} = \frac{{ - 4\sigma^{ * } }}{{3k_{R} }}\frac{{\partial T^{4} }}{\partial y}. $$

where $T$ is heat, $\alpha$ is the coefficient of thermal diffusivity, $Q_{0}$ is dimensional heat production, $(\rho c)_{f}$ is the heat capacity of the liquid, and $(\rho c)_{p}$ is the temperature capacity of the nanoparticles.

The current work assumes slip velocity, thermal and nanoparticles at the surface wall. Therefore, the appropriate boundary conditions can be written as follows:

10 $$ \left. {\begin{array}{*{20}c} {u = cx + L\frac{\partial u}{{\partial y}},\upsilon = 0,T = T_{w} + \beta_{1} \frac{\partial T}{{\partial y}},C = C_{w} + \beta_{2} \frac{\partial C}{{\partial y}},N = 0\;at\;y = 0} \\ {u \to 0,\upsilon \to 0,T \to T_{\infty } ,C \to C_{\infty } ,N \to 0\;as\;y \to \infty } \\ \end{array} } \right\}, $$

where $\beta_{1} ,\beta_{2}$ are the coefficients of heat and mass slip, c is a constant, $cx$ represents the wall velocity and $T_{w} > T_{\infty }$.

### Important physical quantities

The important physical amounts in this analysis are the skin friction parameter**,** $Cf_{y}$ which acts along the $y$ direction, the Nusselt numeral $Nu$ and the Sherwood numeral $Sh$, that are described by Ibrahim^41^ as:

11 $$ Cf_{y} = \frac{{\tau_{w} }}{{\rho (ax)^{2} }},\,\,\,\tau_{w } = \left( {1 - n + k} \right)\frac{\partial u}{{\partial y}} + \frac{n\Gamma }{{\sqrt 2 }}\left( {\frac{\partial u}{{\partial y}}} \right)_{y = 0} , $$

where the skin friction parameter indicates local amount and substantially implies the ratio between the local shear stress to the dynamics pressure, and

12 $$ Nu = \frac{{xq_{w} }}{{k\left( {T_{w} - T_{\infty } } \right)}},\,\,\,q_{w} = - k\left( {\frac{\partial T}{{\partial y}}} \right)_{y = 0} , $$

represents the Nusselt numeral is the ratio between convective and conductive temperature transmission at a border in a liquid. Finally, we have

13 $$ Sh = \frac{{xq_{m} }}{{k\left( {C_{w} - C_{\infty } } \right)}},\,\,\,q_{m} = - k\left( {\frac{\partial C}{{\partial y}}} \right)_{y = 0} , $$

where the Sherwood numeral is specified as the ratio between the convective mass transmission and the mass diffusivity.

### Convenient conversions of relationship

The fundamental nonlinear partial differential equations are transformed into other ordinary ones by an effective similarity conversion. Drawing on the work of Fatunmbi and Okoya^42^ and Ishak^43^, the required similarity transformations can be created as:

14 $$ \left. \begin{gathered} u = U_{w} f^{\prime}(\eta ) = cxf^{\prime}(\eta ),\,\,\,\,\,\,\,\upsilon = - \sqrt {c\nu } f(\eta ),\, \hfill \\ \theta (\eta ) = \frac{{T - T_{\infty } }}{{T_{w} - T_{\infty } }},\,\varphi (\eta ) = \frac{{C - C_{\infty } }}{{C_{w} - C_{\infty } }},\,\,N = cx\,\sqrt {\frac{c}{\nu }} {\text{H(}}\eta )\,\,\,\,\,{\text{and}}\,\,\eta = \,\sqrt {\frac{c}{\nu }} y \hfill \\ \end{gathered} \right\}, $$

where $F(\eta ),\,\theta (\eta ),\,\varphi (\eta )$ and ${\text{H(}}\eta )$ are non-dimensional speed, heat, nanoparticles concentration, correspondingly**,** and $\eta$ is a non-dimensional relationship coordinate.

Under the conversions (11)**,** Eqs. (5–8) may be formulated as:

15 $$ \left( {1 - n + K} \right)f^{{\prime \prime \prime }}  + nWef^{{\prime \prime }} f^{{\prime \prime \prime }}  + ff^{{\prime \prime }}  - \left( {f^{\prime } } \right)^{2}  + \left( {M^{2}  + \frac{1}{{Da}}} \right)f^{\prime }  + KH^{\prime }  = 0, $$

16 $$\left( {1 + \frac{K}{2}} \right)H^{\prime\prime} + K\left( {2H - f^{\prime\prime}} \right) - Hf^{\prime} + fH^{\prime} = 0, $$

17 $$ \left( {\frac{1 + R}{{R\Pr }}} \right)\theta^{\prime\prime} + f\theta^{\prime} + Nb\theta^{\prime}\varphi^{\prime} + Nt\theta^{{\prime}{2}} + M^{2} Ecf^{{\prime}{2}} + Q\theta = 0, $$

18 $$ \varphi^{\prime\prime} + Le\left( {f\varphi^{\prime} + \frac{Nt}{{Nb}}\theta^{\prime\prime} - R_{2} \varphi } \right) = 0. $$

The solutions of these equations are subjected to the border restrictions:

19 $$ \left. \begin{gathered} f^{\prime}(0) = \lambda + \alpha f^{\prime\prime}, \, f(0) = 0, \, \theta (0) = 1 + b_{1} \theta^{\prime},\varphi = 1 + b_{2} \varphi^{\prime},H(0) = 0, \, at \, \eta = 0, \hfill \\ f^{\prime} \to 0, \, \theta \to 0, \, \varphi \to 0,H \to 0, \, \eta \to \infty , \hfill \\ \end{gathered} \right\} $$

where

$$K = \frac{\kappa }{v\rho }$$, $$We = \frac{{\sqrt 2 a\Gamma U_{w} }}{\sqrt \nu }$$, $$M^{2} = \frac{{\sigma B_{0}^{2} (x)}}{\rho c}$$, $$\Pr = \frac{{(\rho c)_{f} \nu }}{\alpha }$$, $$R = \frac{{3k_{R} \alpha (\rho c)_{f} }}{{16\alpha^{*} T_{\infty }^{3} }}$$, $$Nb = \frac{{(\rho c)_{p} }}{{(\rho c)_{f} }}\frac{{D_{B} (C_{\omega } - C_{\infty } )}}{\nu }$$, $$Nt = \frac{{(\rho c)_{p} }}{{(\rho c)_{f} }}\frac{{D_{T} }}{{T_{\infty } \nu (T_{\omega } - T_{\infty } )}}$$, $$Ec = \frac{{\alpha U_{w} }}{{(\rho c)_{f} c}}$$, $$Q = \frac{{Q_{0} }}{{(\rho c)_{f} c}}$$, $$R_{2} = \frac{{R_{1} }}{c}$$, $$Le = \frac{\nu }{{D_{B} }}$$, $$\alpha = \sqrt {\frac{c}{\nu }} L$$, $$b_{1} = \sqrt {\frac{c}{\nu }} \beta_{1}$$, $$b_{2} = \sqrt {\frac{c}{\nu }} \beta_{2}$$ and, $$Da = \frac{cL}{\nu }$$.

## Mathematical technique

The scheme of the governing, fundamental Eqs. (13)–(15) with border restrictions (16) is numerically explained utilizing the shooting technique with the aid of Mathematica 11. For utilizing the method, the governing third important equations are converted to a scheme of first order ones. To guarantee that each numerical value approach asymptotic worth precisely, $\eta_{\infty } = \,6$ is considered. The governing structure of Eqs. (13–15) can be formulated along with the following forms:

20 $$\left. \begin{gathered}   f^{\prime }  = z_{1} ,\;z_{1}^{\prime }  = z_{2} , \hfill \\   {\text{z}}^{\prime } _{2}  = \left( {{\text{z}}_{1}^{2}  - f{\text{z}}_{2}  + (M + \tfrac{1}{{Da}}{\text{)z}}_{1}  - {\text{Kz}}_{3} } \right)/\left( {\left( {1 - n + K} \right) + nWe{\text{ z}}_{2} } \right), \hfill \\   H^{\prime }  = z_{3} ,z_{3}^{\prime }  = \left( {{\text{z}}_{1} {\text{g}} - f{\text{z}}_{3}  - {\text{K(2g  +  z}}_{2} )} \right)/\left( {1 + \tfrac{{\text{K}}}{2}} \right) \hfill \\   \theta ^{\prime }  = z_{4} ,z_{4}^{\prime }  =  - \left( {\tfrac{{{\text{RPr}}}}{{1 + {\text{R}}}}} \right)\left( {f{\text{ z}}_{4}  + Nbz_{4} {\text{z}}_{5}  + Nt{\text{ z}}_{4}^{2}  + M^{2} Ecz_{1}^{2}  + {\text{Q}}\theta } \right) \hfill \\   \varphi ^{\prime }  = z_{5} ,z_{5}^{\prime }  =  - Le\left( {fz_{5}  + \tfrac{{Nt}}{{Nb}}z_{4}^{\prime }  - R_{2} \varphi } \right) \hfill \\  \end{gathered}  \right\}$$

Then, we solve the ODEs with the initial conditions given by

21 $$ \left. \begin{gathered} f(0) = 0, \hfill \\ f^{\prime}(0) = \lambda + \alpha f^{\prime\prime}(0), \hfill \\ H(0) = 0, \hfill \\ \theta (0) = 1 + b_{1} \theta^{\prime}(0) \hfill \\ \varphi (0) = 1 + b_{2} \varphi^{\prime}(0) \hfill \\ \end{gathered} \right\}. $$

The conditions at the regular limits (21) are not adequate to obtain the solutions of the combined system (20), so primary guesstimates for $f^{\prime\prime}(0)$, $\theta^{\prime}(0)$, $H^{\prime } (0)$ and $\varphi^{\prime } (0)$, which expressed by $z^{\prime}_{1} (0)$,$z_{4} (0)$, $z_{3} (0)$ and $z_{5} (0)$, respectively are automatically suggested. First, the solutions begin in the location of $\eta = 10^{ - 4} \,$ to avoid the singularity at $\eta = 0\,$. The reasonable supposition values for $f^{\prime\prime}(0)$,$\theta^{\prime}(0)$, $H^{\prime } (0)$ and $\varphi^{\prime } (0)$ are picked by the shooting technique, and then the integration process is completed. By Mathematica Software Version 11.0.0.0, the Runge–Kutta method is functioning, and the numerical solutions are attained. If the attained solution does not meet the acceptable range of convergence, then the primary guesses are re-suggested and the procedure is recurrent until the solution satisfies the convergence measure. Moreover, we compare the estimated amounts of $f^{\prime}$, $\theta$, $\varphi$ and $H$ at $\eta = 6$ (as infinity value) as well as the specified boundary conditions $f^{\prime}(6) = 0$,$\theta (6) = 0$, $\varphi (6) = 0$ and $H(6) = 0$, then modify the values of $f^{\prime\prime}(0)$, $\theta^{\prime}(0)$, $H^{\prime } (0)$ and $\varphi^{\prime } (0)$ to get more iterations for solutions with further accuracy.

## Findings and interpretation

A stationary, non-Newtonian nanofluid in the vicinity of a stretching surface, obeying the tangent hyperbolic prototype, is addressed. The model is influenced by a normal uniform magnetic field to the sheet. Heat and nanoparticles mass transfer is taken in account with Ohmic dissipation, temperature source, thermal radiation, and chemical response influences. The non-dimensional fundamental Eqs. (4)–(8) with the convenient border restriction (10) are numerically examined by processing the Runge–Kutta and Shooting method.

To substantially explain the problem, the findings are examined to exhibit the impacts of the restriction factors on the physical distributions. These factors incorporate the Weissenberg factor $We$, the power law factor $n$, the vortex viscosity factor $K$, the magnetic factor $M$, the stretching parameter $\lambda$, the Darcy numeral $Da$, the Prandtl numeral $\Pr$, the Eckert numeral $Ec$, the radiation factor $R$, the thermophoresis factor $N_{T}$, and the Brownian movement factor $N_{B}$. The study at hand concentrates on the influences of the limitations on speed, heat, nanoparticles distributions. These profiles are plotted in accordance with the data mentioned in Figs. 2–27.

**Figure 2:**
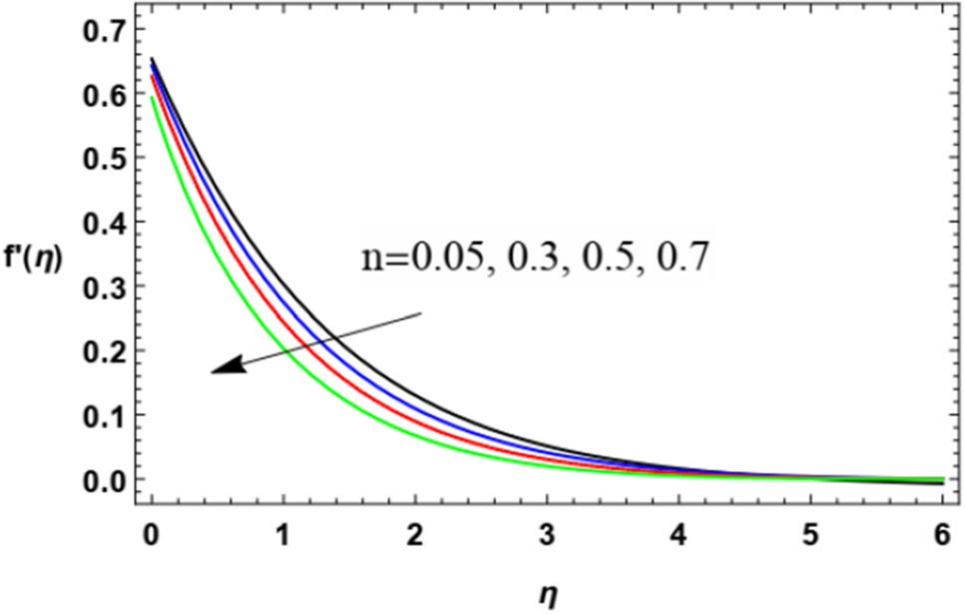
Variation of the radial velocity $f^{\prime } (\eta )$ versus $\eta$ as given in Eq. (15) to depict the effect of power law index $n$.

### Velocity distribution

**Figure 3:**
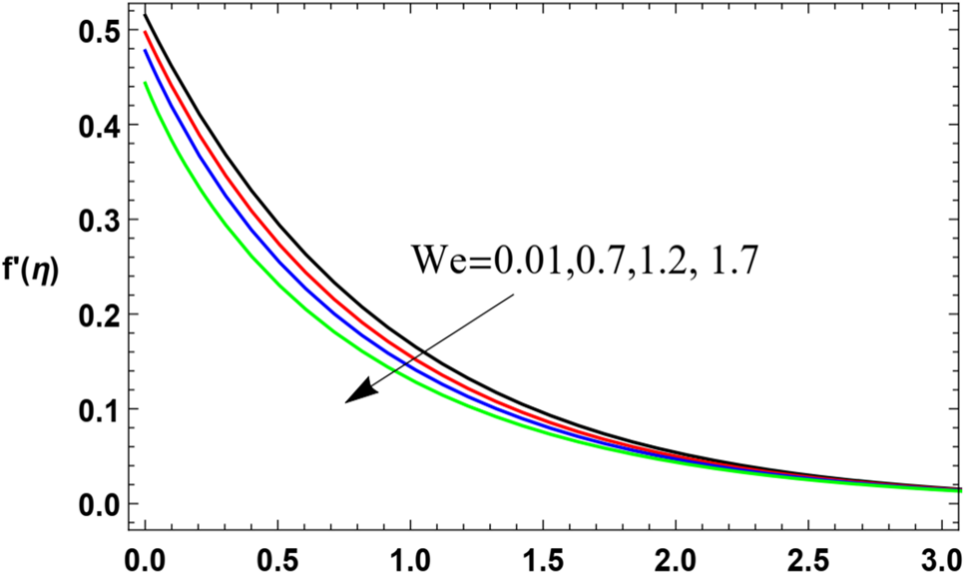
Variation of the radial velocity $f^{\prime } (\eta )$ versus $\eta$ as given in Eq. (15) to depict the effect of parameter of the Weissenberg $We$.

**Figure 4:**
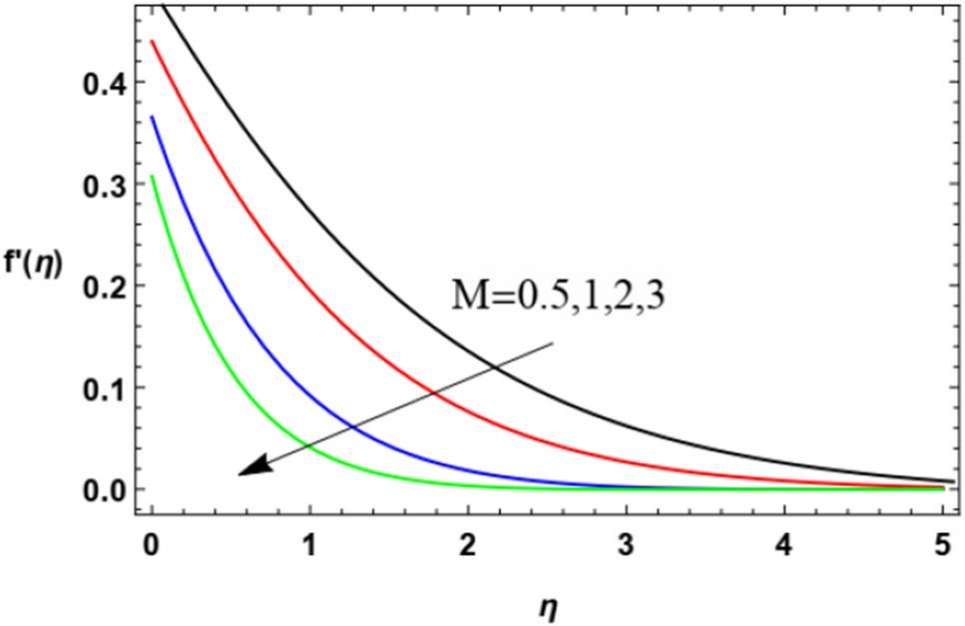
Deviation of the radial speed $f^{\prime } (\eta )$ versus $\eta$ as given in Eq. (15) to depict the effect of the magnetic parameter $M$.

**Figure 5:**
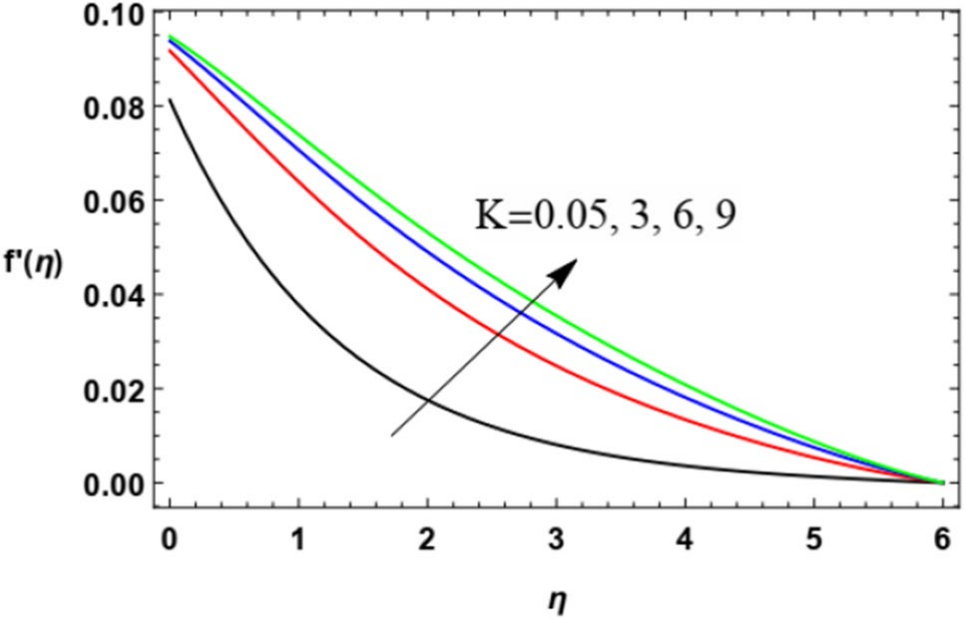
Deviation of the radial speed $f^{\prime } (\eta )$ versus $\eta$ as given in Eq. (15) to depict the effect of the material parameter $K$.

**Figure 6:**
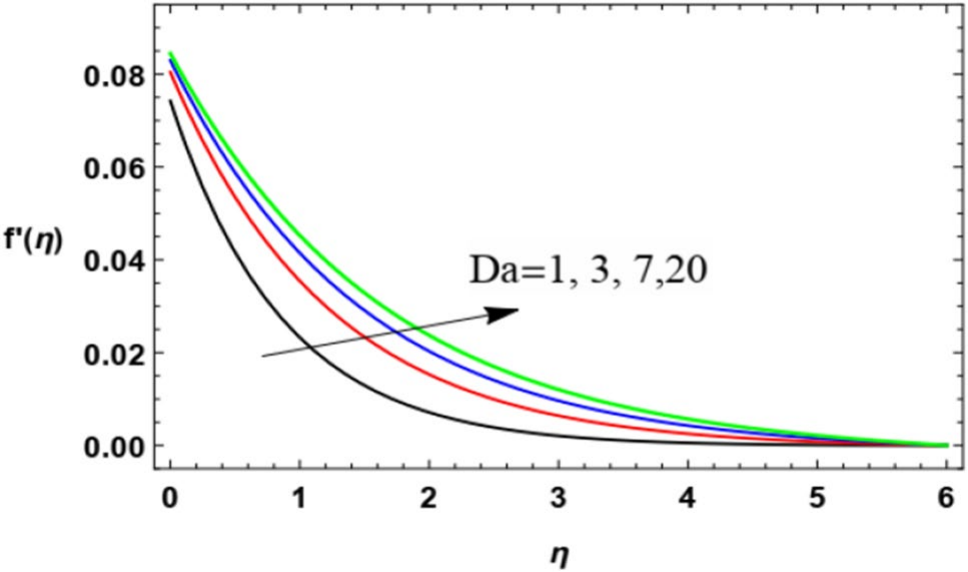
Deviation of the radial speed $f^{\prime } (\eta )$ versus $\eta$ as given in Eq. (15) to illustrate the influence of Darcy numeral $Da$.

**Figure 7:**
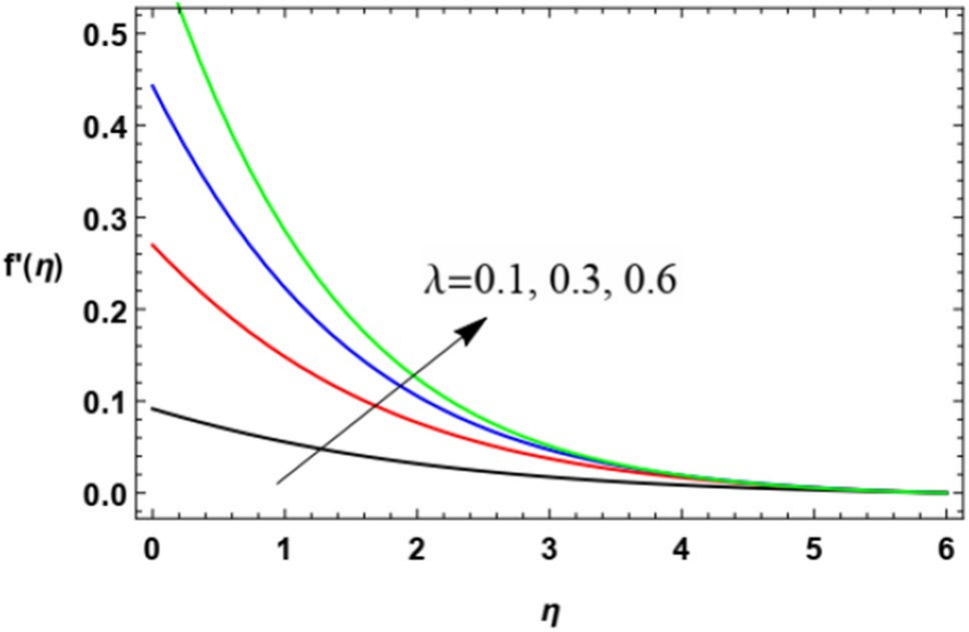
Deviation of radial speed $f^{\prime } (\eta )$ versus $\eta$ as given in Eq. (15) to depict the effect of the stretching parameter $\lambda$.

The non-dimensional radial speed $u$ is mapped against the non-dimensional parameter $\eta$ through Figs. 2, 3, 4, 5, 6 and 7 to illustrate the influences of the proper parameters that appear in this problem. It is seen that reduction of the radial speed is a general performance with the whole of $\eta$ i.e., far away from the wall. Figures 2, 3 and 4 demonstrate the impacts of three different parameters on the fluid speed, namely, the power law parameter $n$, the Weissenberg parameter $We$ and the magnetic field parameter $M$. As seen from Fig. 2, the rise of the power law parameter decreases the flow velocity, which reduces the hydraulic boundary area of the fluid. Materially, the growth of $n$ leads to a rise in the fluid viscosity, which leads to a weak motion of the flow. This result accords with the works of Ibrahim^32^, and Hussain et al.^44^. The same behavior corresponds to $We$ as shown in Fig. 3. Physically, the Weissenberg parameter represents the relaxation coefficient of the fluid. Moreover, the Weissenberg numeral defines the ratio between the elastic and viscous forces. Consequently, the rise of $We$ means more elasticity of fluid, which implies that the growth of $We$ yields a reduction in the fluid speed. The same result was concluded by Ibrahim^32^, and Hussain et al.^44^. Accordingly, the impact of the magnetism constraint $M$ on the flow speed appears in Fig. 4, where the rise of the magnetic strength waves, as a measure of the Lorentz force, indicates a drop in the fluid velocity. Physically, the Lorentz power impedes the fluid flow and tends to be more prevailing with the rise of $M$, which causes a drop in the fluid velocity. This finding corresponds to that described by Zakir Ullah et al.^36^ and Akbar et al.^45^. Figure 5 signifies the impact of the vortex viscosity factor $K$ on the speed outline. It is found that the rise in the microrotation parameter leads to a rise in velocity. From the physical standpoint, microrotation means the rotation of the microscopic parts of a fluid, crystal etc. Subsequently, the rise of these rotations accelerates the fluid flow, and hence increases velocity. This result corresponds to that described in the earlier works of Seddek et al.^46^ and Javed et al.^47^.

Figures 6 and 7 display the behavior of the velocity profile with $\eta$ coordinate for various values of the Darcy numeral $Da$ and the stretching factor $\lambda$. Figure 6 shows that the rise in Darcy number yields an increase in the fluid speed. Actually, the Darcy numeral depends on the permeability of the medium, where the Darcy numeral represents the ratio between the permeability of the medium and its cross-sectional area so the rise of $Da$ means a growth of the permeability of the medium and in turn a rise in the speed of the flow, so such influence turns out. This result is consistent with those earlier concluded in Ref.^48^.

On the other hand, in Fig. 7, it is noticed that the rise in the expanding factor $\lambda$ results in a rise in the fluid velocity. Physically, the growth of the walls stretching coefficients helps the flow to move easily in the movement direction, hence this velocity component increases with the rise of $\lambda$. This result is consistent with the same outcome as given in Zakir Ullah et al.^36^.

### Microrotation (Spin) Velocity distribution

**Figure 8:**
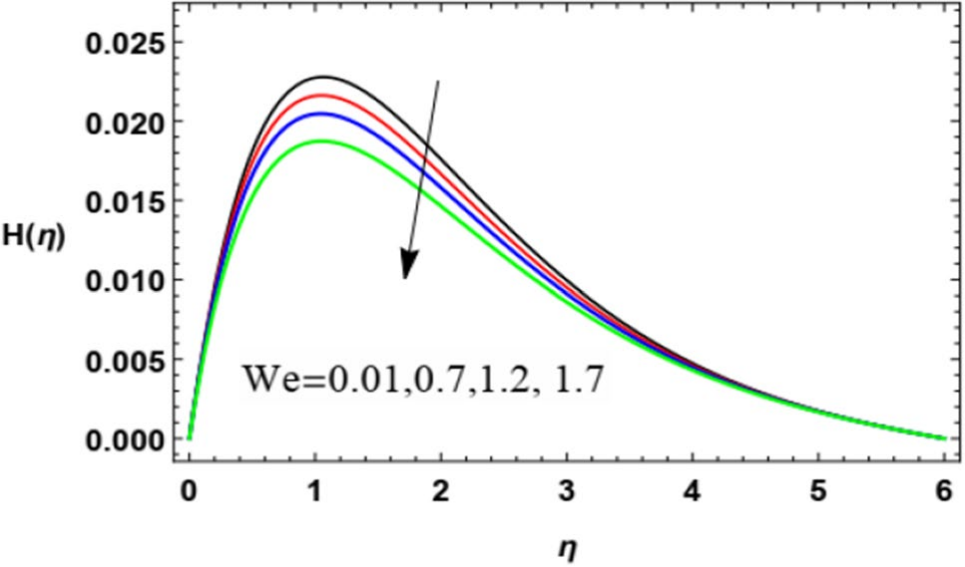
Deviation of the microrotation profile $H(\eta )$ versus $\eta$ as given in Eq. (16) to depict the effect of parameter of the Weissenberg $We$.

**Figure 9:**
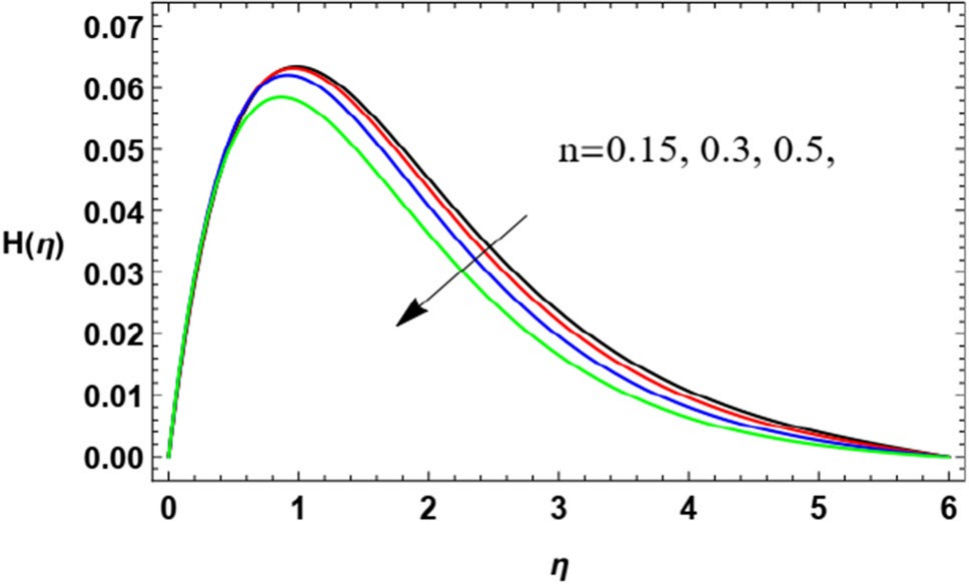
Deviation of the microrotation profile $H(\eta )$ versus $\eta$ as given in Eq. (16) to depict the effect of power law factor $n$.

**Figure 10:**
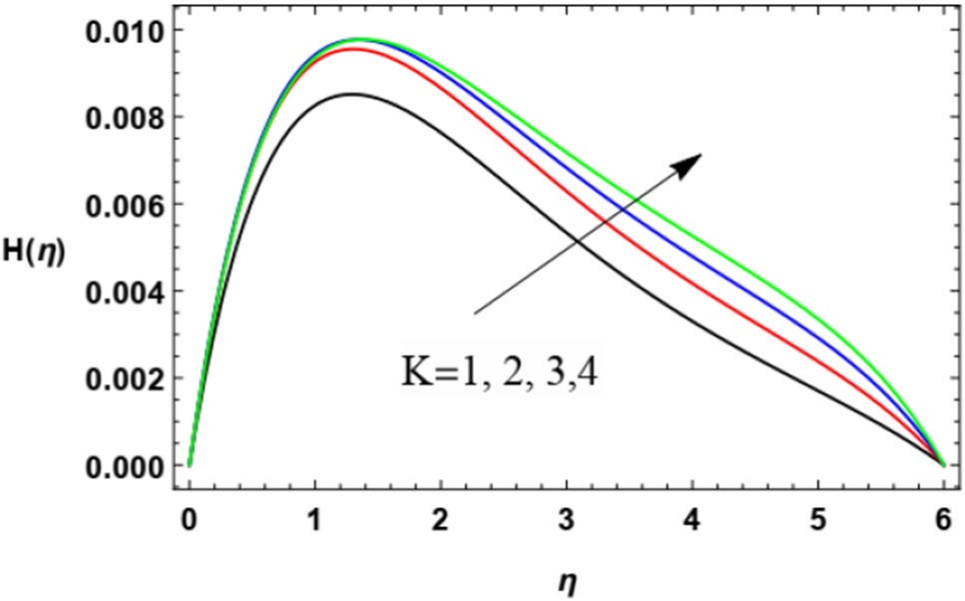
Variation of the microrotation profile $H(\eta )$ versus $\eta$ as given in Eq. (16) to illustrate the influence of the material factor $K$.

**Figure 11:**
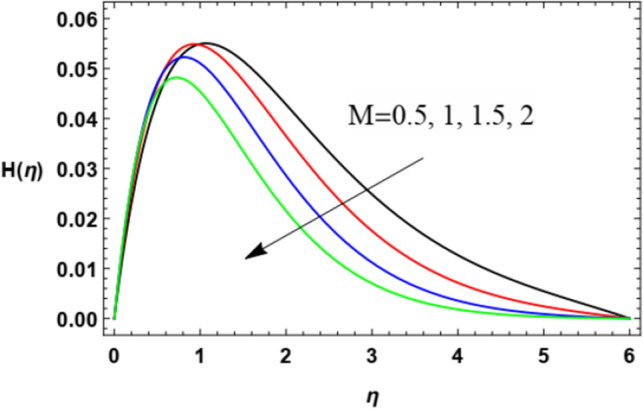
Variation of the microrotation profile $H(\eta )$ versus $\eta$ as given in Eq. (16) to illustrate the influence of the magnetic factor $M$.

**Figure 12:**
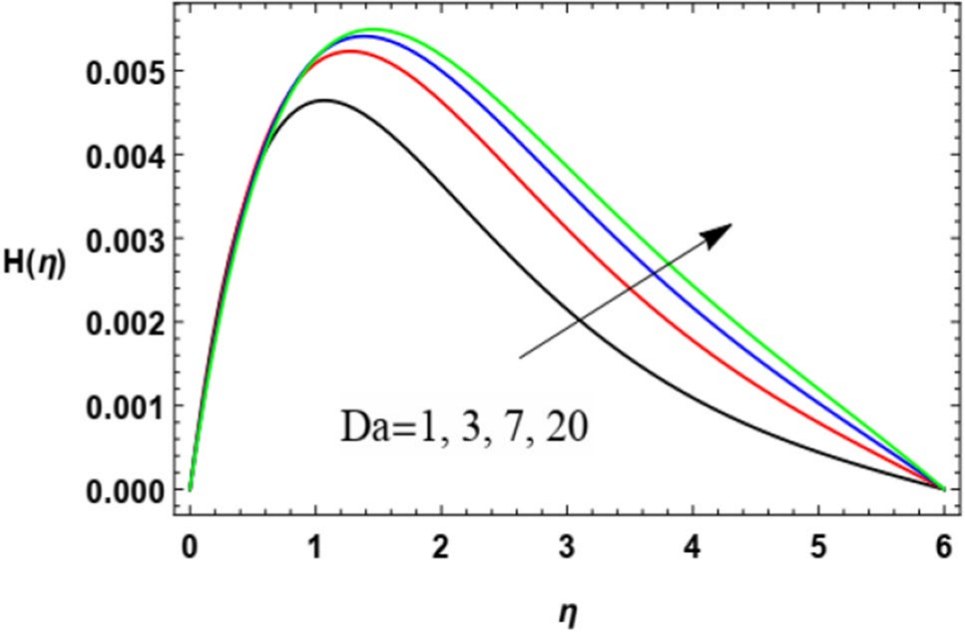
Variation of the microrotation profile $H(\eta )$ versus $\eta$ as given in Eq. (16) to depict the effect of Darcy number $Da$.

**Figure 13:**
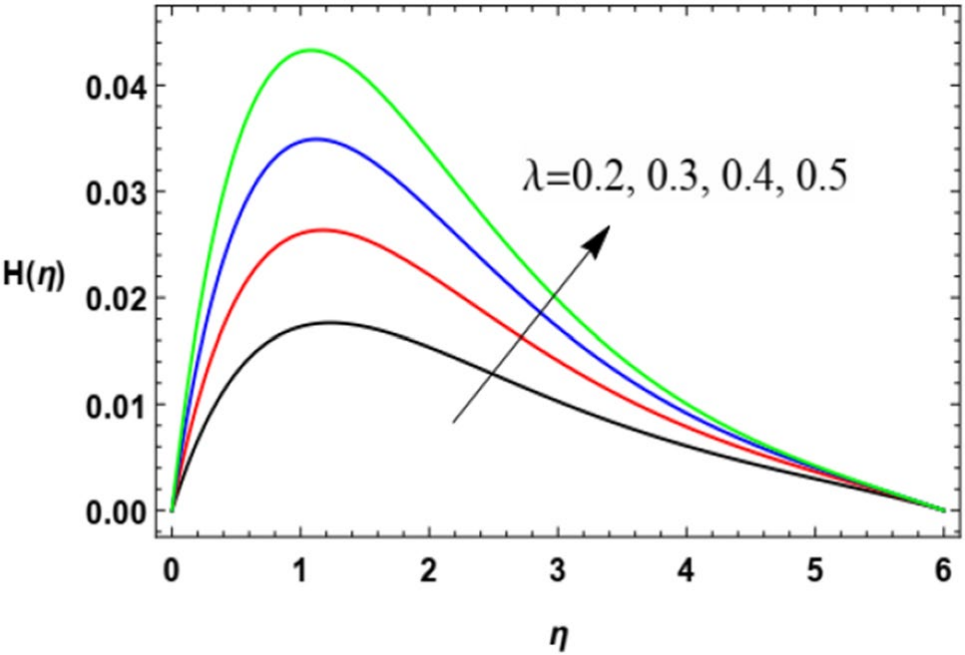
Variation of the microrotation profile $H(\eta )$ versus $\eta$ as given in Eq. (16) to illustrate the influence of the stretching factor $\lambda$.

With a view to clarify the influences of the relevant parameters on the microrotation (spin or angular) velocity $H$, Figs. 8, 9, 10, 11, 12 and 13 are outlined. By these diagrams, the microrotation speed $H$ is graphed against the dimensionless parameter $\eta$. As noted, the microrotation distribution noticeably increases until some values of $\eta \cong 1$ after which the behavior is reversed and decreases rapidly. Figures 8 and 9 denote the impacts of the power parameter $n$ and the Weissenberg parameter $We$ on the microrotation velocity profile. These two figures show an opposite behavior for the values of these parameters with the behavior of the microrotation velocity, where the rise in the values of these factors leads to a decrease in the microrotation velocity profile after a period of consistency near the wall. It is noted that these effects are the same as those of these parameters on the fluid radial velocity and have the same physical explanations. These findings are in accord with those given by Zakir Ullah et al.^36^ and Ishak^43^.

Figures 10 and 11 demonstrate the impacts of $K$ and $M$ on the microrotation velocity. As shown from Fig. 10, the increase of the vortex viscosity parameter $K$ increases the microrotation velocity. Given that the microrotation represents the rotation of microscopic parts of a fluid, then the growth of these rotations accelerates the fluid angular velocity. This result is in accord with that concluded in Javed et al.^47^. On the contrary, the growth in the magnetism factor $M$ increases the Lorenz force that impedes the movement of the flow whether in the radial direction, as seen previously in Fig. 4, or in the angular direction as shown by Fig. 11. These results are found to be consistent with those of Zakir Ullah et al.^36^, Akbar et al.^45^, and Ahmad et al.^49^.

Figures 12 and 13 show the behavior of the angular velocity $H$ for different values of the Darcy numeral $Da$ and the stretching factor $\lambda$, correspondingly. It is obvious from Fig. 6 that the angular velocity rises with the growth of Darcy number. As observed above, The Darcy numeral signifies the proportion between the permeability of the medium and its cross-sectional area so the rise of $Da$ means a growth of the permeability of the medium and making the flow much easier hence increases the velocity values. From Fig. 13, one can notice that the spin speed also rises with the rise of the stretching factor $\lambda$. The physical interpertation of this effect of $\lambda$ has been mentioned above.

### Temperature distribution

**Figure 14:**
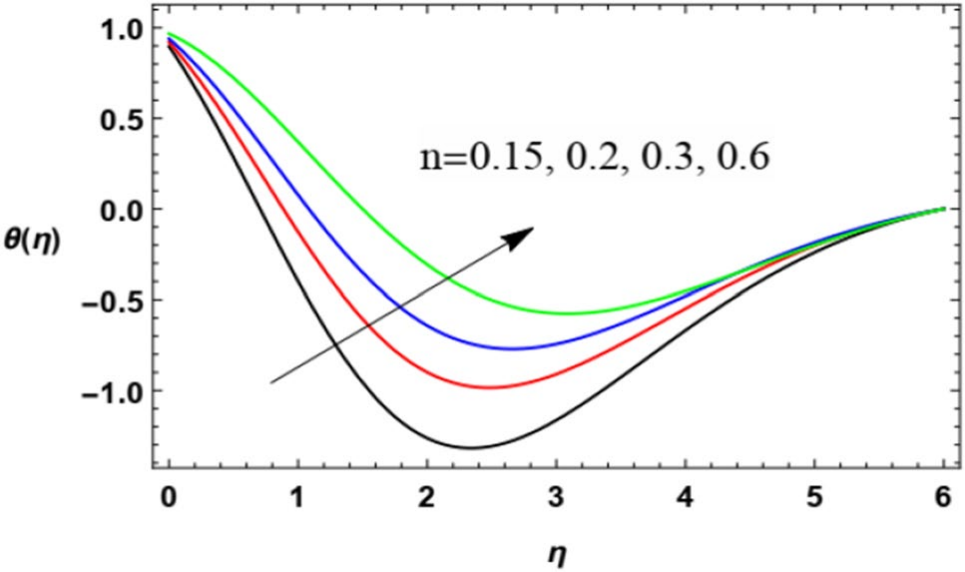
Deviation of the temperature profile $\theta (\eta )$ against $\eta$ as given in Eq. (17) to illustrate the influence of the power law factor $n$.

**Figure 15:**
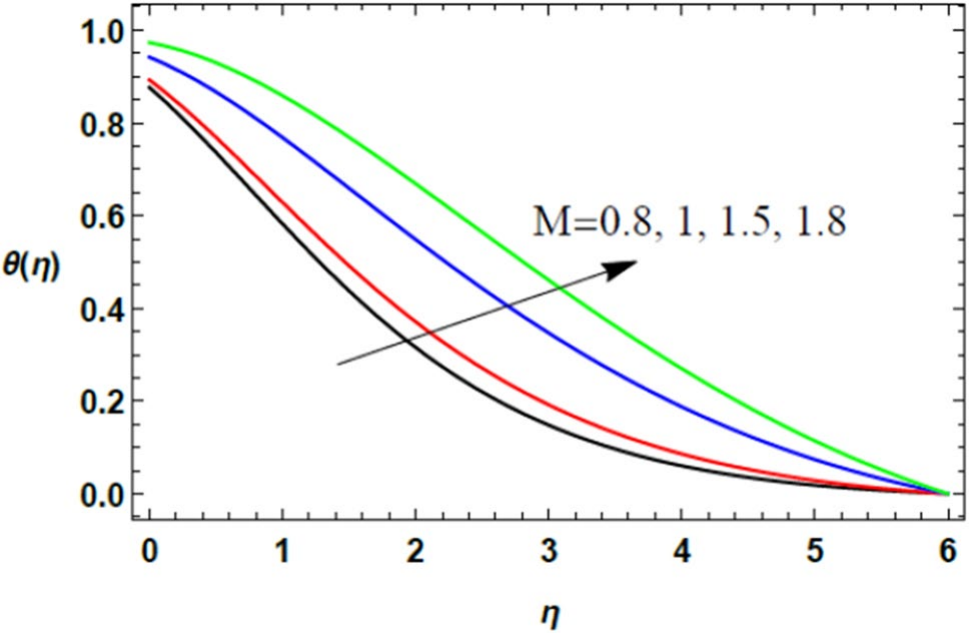
Deviation of the heat profile $\theta (\eta )$ against $\eta$ as given in Eq. (17) to illustrate the influence of the magnetic factor $M$.

**Figure 16:**
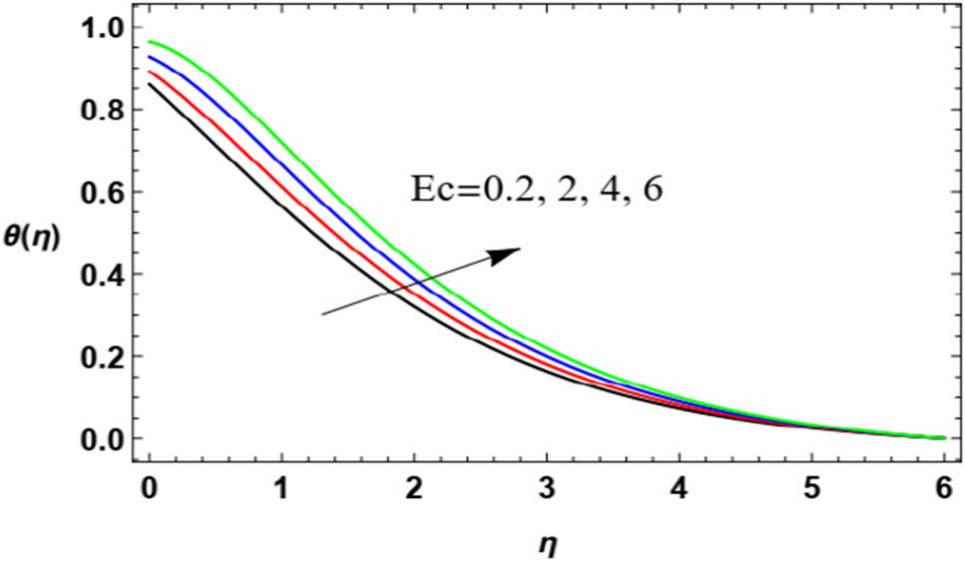
Deviation of the heat profile $\theta (\eta )$ versus $\eta$ as given in Eq. (17) to illustrate the influence of Eckert numeral $Ec$.

**Figure 17:**
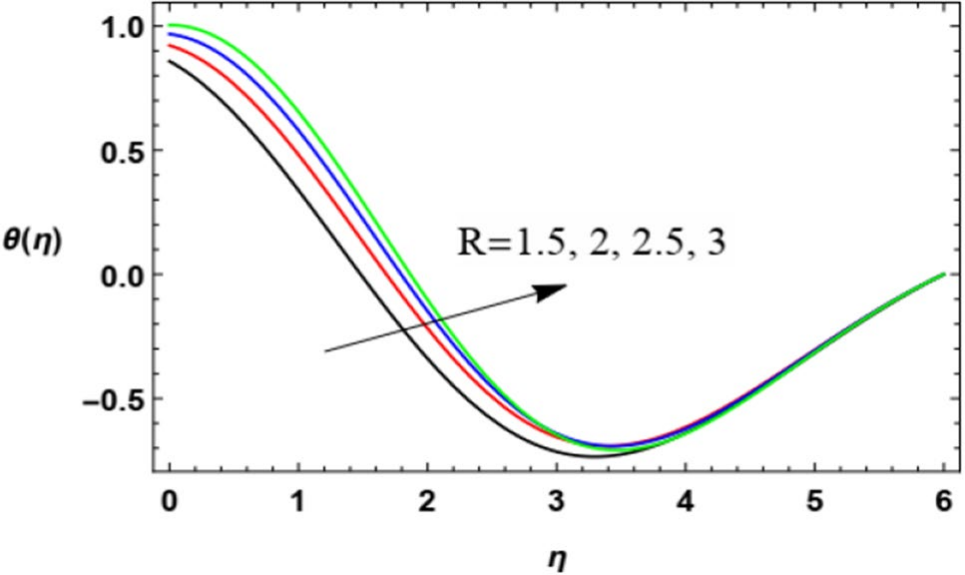
Deviation of the heat profile $\theta (\eta )$ versus $\eta$ as given in Eq. (17) to illustrate the impact of the Thermal radiation factor $R$.

**Figure 18:**
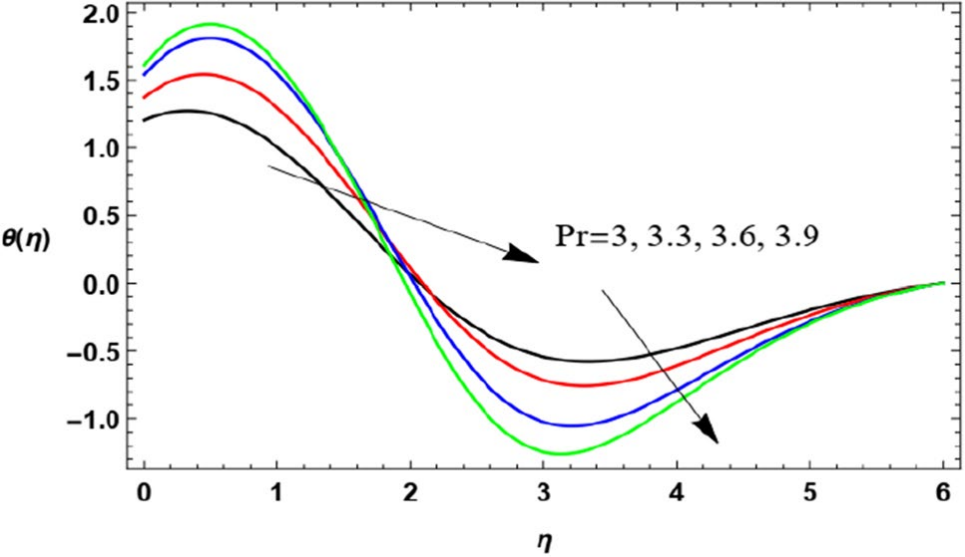
Deviation of the heat profile $\theta (\eta )$ versus $\eta$ as given in Eq. (17) to illustrate the influence of the Prandtl numeral $\Pr$.

**Figure 19:**
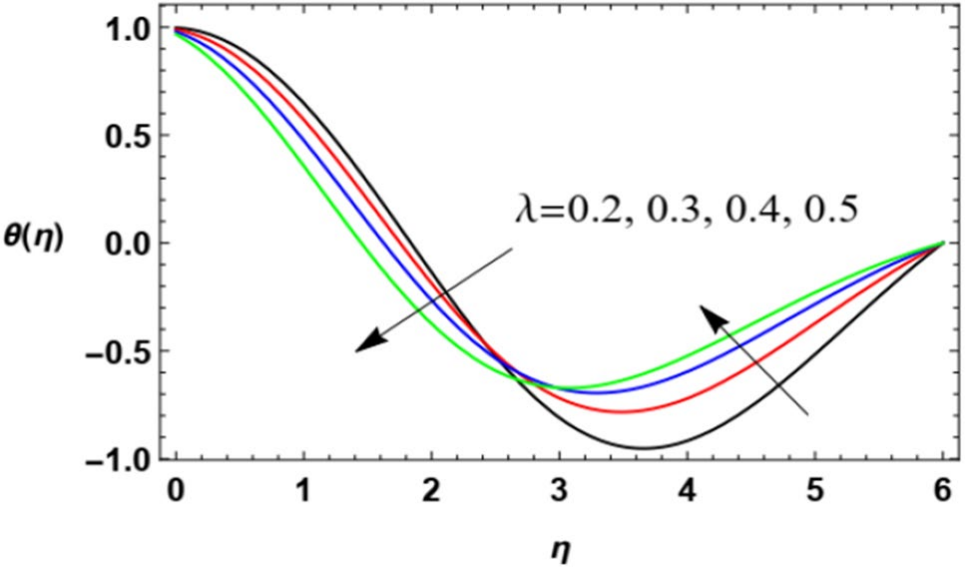
Variation of the temperature distribution $\theta (\eta )$ versus $\eta$ as given in Eq. (17) to illustrate the influence of the stretching factor $\lambda$.

**Figure 20:**
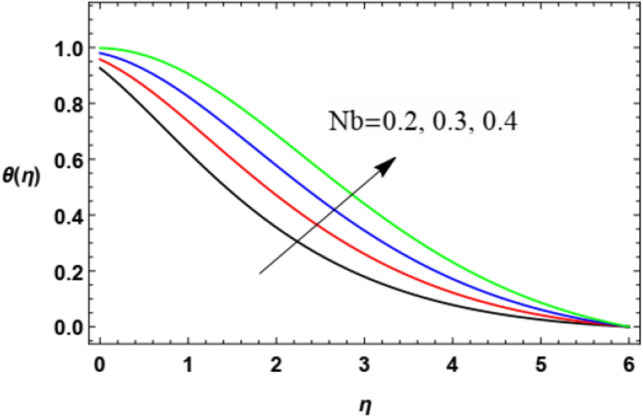
Variation of the temperature distribution $\theta (\eta )$ versus $\eta$ as given in Eq. (17) to illustrate the influence of the Brownian motion factor $Nb$.

**Figure 21:**
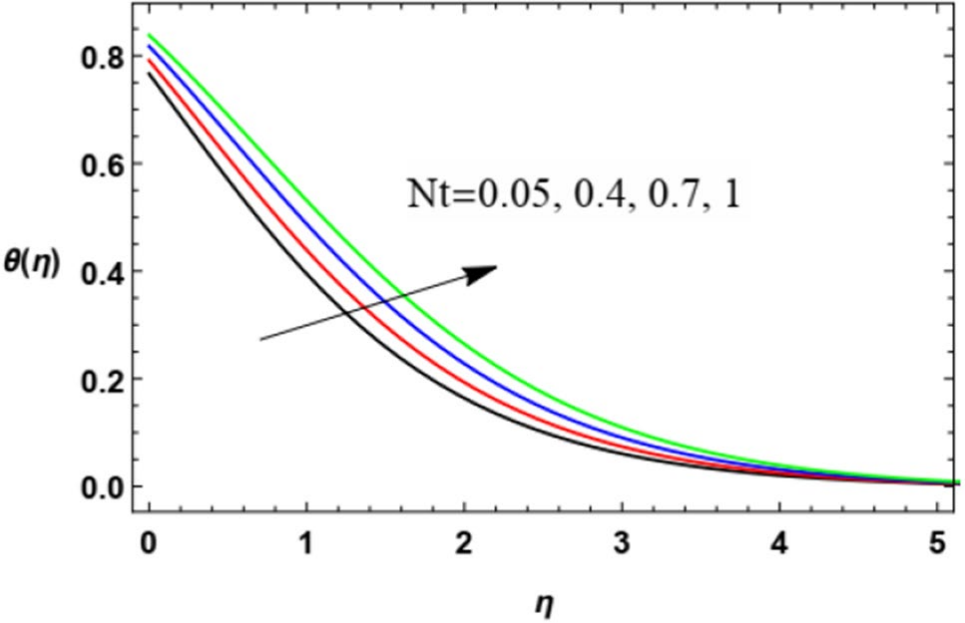
Deviation of the heat profile $\theta (\eta )$ versus $\eta$ as given in Eq. (17) to illustrate the influence of the thermophoresis factor $Nt$.

Figures 14, 15, 16, 17, 18, 19, 20 and 21 demonstrate the non-dimensional temperature distribution $\theta$ versus the non-dimensional variable $\eta$ to clarify the impacts of the power law parameter $n$, the magnetism factor $M$, the Eckert numeral $Ec$, the radiation parameter $R$, the Prandtl numeral $\Pr$, the stretching parameter $\lambda$, the Brownian movement factor $N_{B}$, and the thermophoresis factor $N_{T}$.

Figures 14 and 15 illustrate the influences of the power index parameter $n$ and the magnetic parameter $M$ on the heat profile. These two figures show that temperature transmission improves with the rise of both $n$ and $M$. Actually, the increase of $n$ slows down the fluid speed as shown previously in Fig. 2 due to the growth in the fluid viscosity, which in turn increases the fluid temperature. Moreover, the increase of $M$ increases the Lorentz force and slows down the fluid flow, then the temperature grows. The physical explanations of these two effects are as mentioned above in speed distribution. Similar results were found in previous studies by Zakir Ullah et al.^36^, and Akbar et al.^45^.

Figures 16 and 17 are designed to label the performance of the heat profile $\theta (\eta )$ in addition to the non-dimensional align $\eta$ and under the impacts of both the Eckert numeral $Ec$ and the thermal radiation $R$. As shown in Fig. 16, the increase of $Ec$ increases heat transmission. Materially, the Eckert numeral $\mathrm{Ec}$ $Ec$ indicates the structure joining the kinetic energy and the boundary sheet enthalpy change; it also defines heat transmission dissipation. This temperature dissipation produces temperature due to the collaboration of the concerning liquid particles, which leads to a rise in the basic liquid temperature. so its increase naturally produces a rise in the heat of the fluid layer. In Fig. 17, it is found that the rise in the heat radiation factor $R$ intensifies the fluid heat. Acutally, the radiation is one of the heat sources that increases or leaks heat from the current medium. Here, the radiation leads to an increase in heat, which means that it is one of the aspects that activate heat transfer, and therefore it is of practical importance in several fields. The high radiation load of the fluid leads to an increase in its temperature. These results are found to be in accord with the works of El-Dabe et al.^48^, and Abou-zeid^12^.

Figures 18 and 19 show temperature distribution with the $\eta$-coordinate under the influence of different values of $\Pr$ and $\lambda$, where the heat distribution of the fluid increases with the increase of $\Pr$ until a certain point ($\eta \approx 2$) after which the effect is reversed, where the increase of $\Pr$ decreases temperature. Physically, the Prandtl numeral characterizes the proportion of momentum diffusivity (kinematic viscosity) and thermal diffusivity, so it is normal that the rise in the Prandtl numeral leads to a reduction in thermal diffusion. It seems that this is realized, but after a period of flowing away from the surface. After the reflection change point ($\eta \approx 2$), this result agrees with the work of Ahmad et al.^49^. On the contrary, temperature distribution decreases with increase of the stretching parameter $\lambda$ until a certain point ($\eta \approx 3.1$) after which the effect is inverted. As said before, the growth of the walls stretching coefficients helps the flow to be easier, and hence reduces the temperature of the fluid. The last result before the reflection change point ($\eta \approx 3.1$) corresponds to that obtained by Zakir Ullah et al.^36^.

Figure 20 and 21 demonstrate the effects of the Brownian movement factor $Nb$ and thermophoresis factor $Nt$ on heat transmittion. It is noticed in Figs. 20 and 21 that the increase in the Brownian movement factor $Nb$ and the thermal transfer factor $Nt$ increases heat transmission. Materially, the thermophoresis factor $Nt$ enhances the drive of nanoparticles from the hot plate to the adjacent liquid, which yields a rise in the temperature in the nearby liquid as observed in Fig. 20. Similarly, this is because of the way that the thermophoretic force produced by the heat slope makes a quick stream away from the extending surface. By this manner more heated liquid is gotten away from the surface. Furthermore, the rise in the Brownian motion parameter $Nb$, which is considered as a measure of the accidental motion of the nanoparticles, improves the temperature in the zone layers of fluid as shown in Fig. 21. These findings correspond to the works of Shravani et al.^33^, Awais et al.^50^, Gbadeyan^51^, Nadeem et al.^52^, and Ramesh et al.^53^.

### Nanoparticle volume fraction distribution

**Figure 22:**
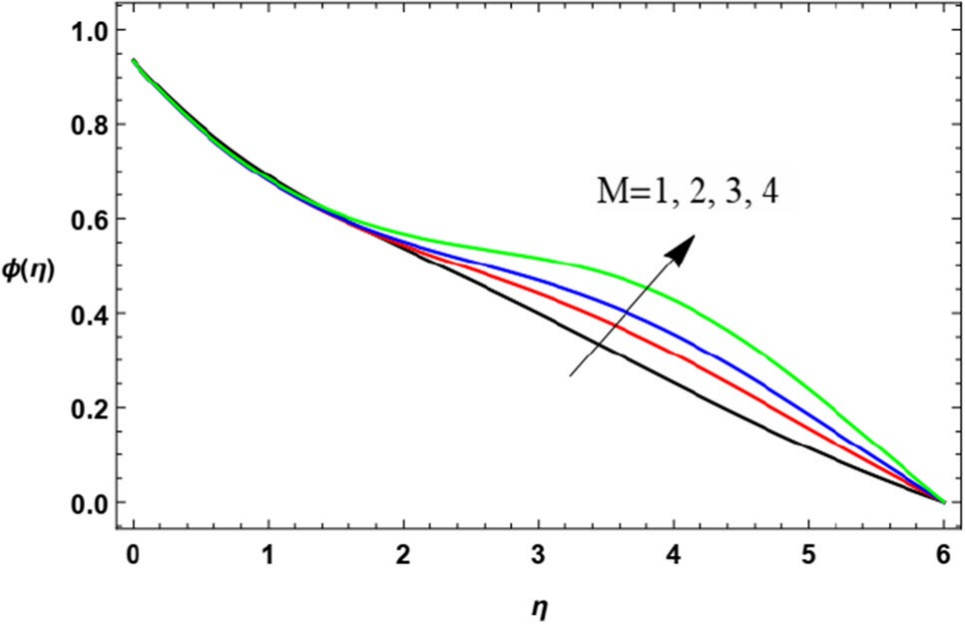
Deviation of the nanoparticle concentration $\varphi (\eta )$ versus $\eta$ as given in Eq. (18) to illustrate the influence of the magnetic factor $M$.

**Figure 23:**
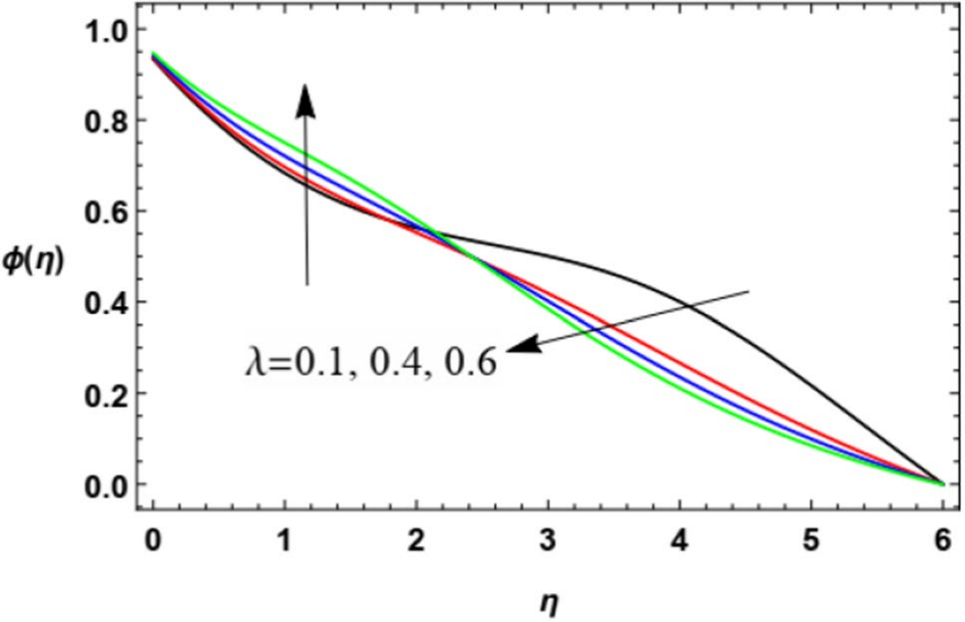
Deviation of the nanoparticle concentration $\varphi (\eta )$ versus $\eta$ as given in Eq. (18) to illustrate the influence of the stretching factor $\lambda$.

**Figure 24:**
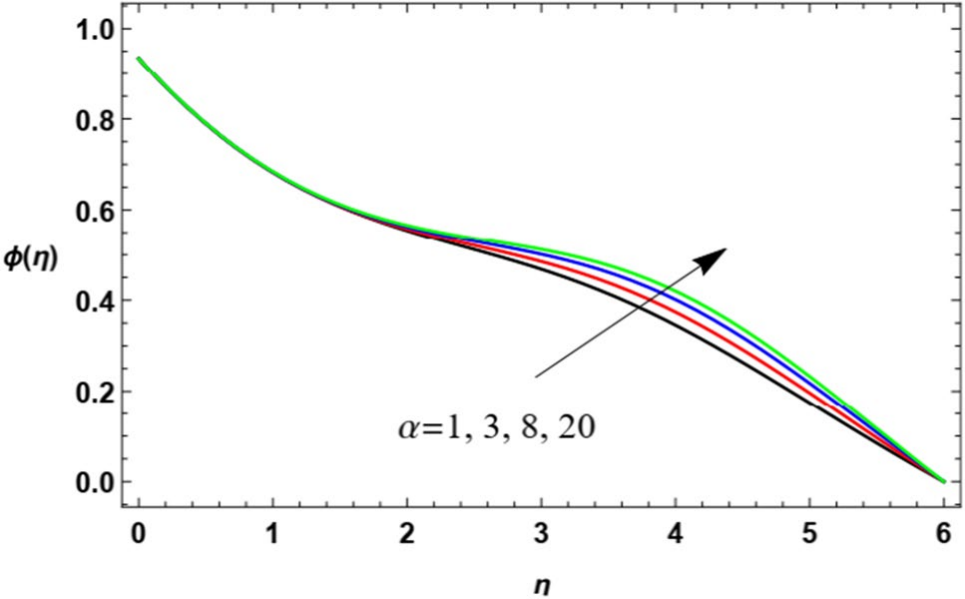
Variation of the nanoparticle concentration $\varphi (\eta )$ versus $\eta$ as given in Eq. (18) to depict the effect of the coefficient of thermal diffusivity $\alpha$.

**Figure 25:**
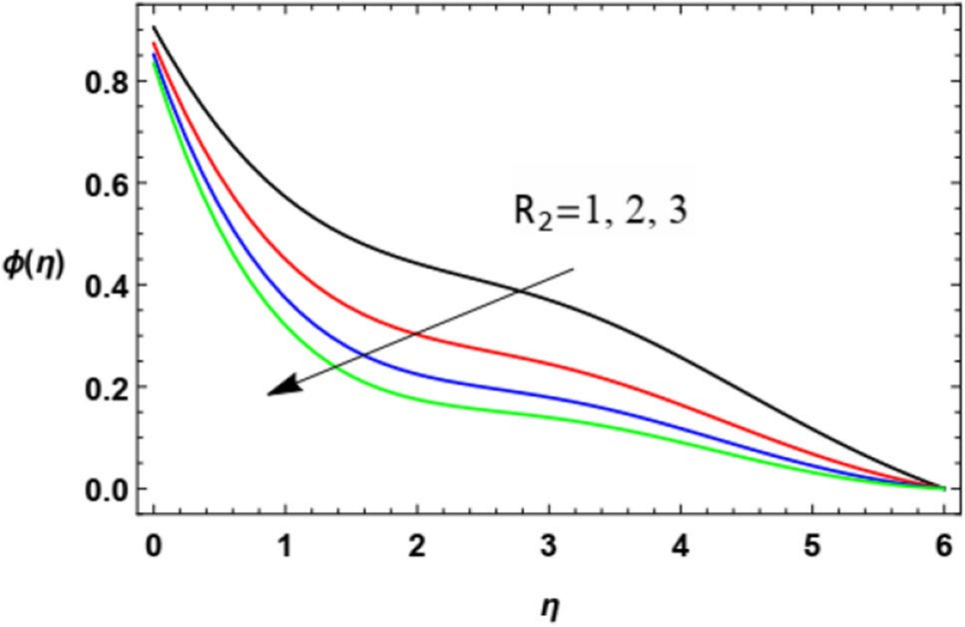
Variation of the nanoparticle concentration $\varphi (\eta )$ versus $\eta$ as given in Eq. (18) to illustrate the influence of Chemical reaction $R_{2}$.

**Figure 26:**
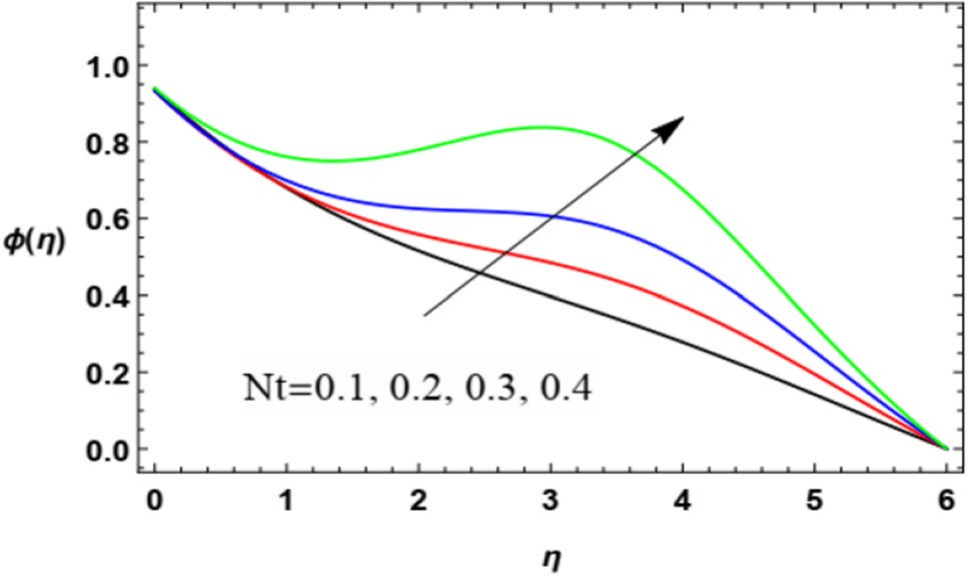
Deviation of the nanoparticle concentration $\varphi (\eta )$ versus $\eta$ as given in Eq. (18) to illustrate the influence of the thermophoresis factor *Nt*.

**Figure 27:**
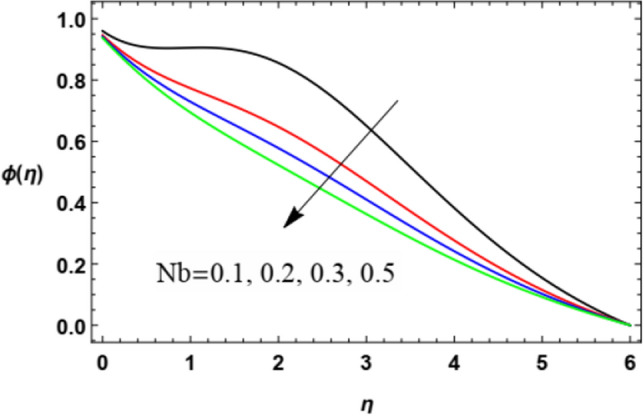
Deviation of the nanoparticle concentration $\varphi (\eta )$ versus $\eta$ as given in Eq. (18) to illustrate the influence of Brownian motion factor *Nb*.

For discussing the influences of the magnetism factor $M$, the stretching parameter $\lambda$, the coefficient of thermal diffusivity $\alpha$, the Chemical reaction $R_{2}$ the thermophoresis factor $Nt$ and the Brownian movement factor $Nb$, on the nanoparticles concentration $\varphi (\eta )$, the solution of Eq. (18) is numerically discussed and plotted through Figs. 22, 23, 24, 25, 26 and 27.

In Fig. 22, it can be noticed that at first, the effect is stable to some extent, but after a while the rise of the magnetism factor $M$ increases the nanoparticles concentration $\varphi (\eta )$. As seen before, the rise of the magnetic parameter decreases the velocity magnitude in the border region due to the enhancement of Lorentz force, hence, the reduction of the velocity in the boundary stratum encourages the accumulation of the nanoparticles diffusion near the border. The same result was obtained in Refs.^37^ and^54^.

Figure 23 shows that there is a dual role of the expanding factor $\lambda$ in the nanoparticles concentration $\varphi (\eta )$. The increase of the expanding parameter initially increases the nanoparticles concentration $\varphi (\eta )$ until $\eta \approx 2.5$ after which the nanoparticles concentration decreases. It can be noticed that this effect is opposite to those on heat transfer, so this is logical because as the temperature rises, the nanoparticles concentartion decreases and vica versa. This result is in accord with the same one concluded in Kitetu et al.^55^.

Figure 24 shows that the nanoparticles volume fraction $\varphi (\eta )$ increases as the thermal diffusivity parameter $\alpha$ rises, as the further stream goes away from the boundary. Physically, thermal diffusivity equals thermal conductivity, divided by density and the specific heat capacity at uniform pressure. It measures the ratio between the ability of a material to conduct thermal energy and its ability to store heat energy. This means that as $\alpha$ increases, the ability of accumulating energy decreases, which leads to loss in temperature and increases the concentration of nanoparticles.

Figure 25 demonstrates the influence of various values of the chemical reaction parameter $R_{2}$ on the nanoparticles concentration $\varphi (\eta )$. It is seen that the nanoparticles concentration decreases with the increases of $R_{2}$. Physically, as $R_{2}$ increases, a wide-ranging dispersion of mass over the surrounding fluid rises. Hence, this increase of $R_{2}$ causes nanoparticles to spread more away over the flow and indicates a drop in the nanoparticle concentration. This result is the same as that obtained by Moatimid et al.^56^.

Figure 26 and 27 depict the impact of the thermophoresis factor $Nt$ and Brownian movement factor $Nb$ on the nanoparticle’s concentration $\varphi (\eta )$. These diagrams show that the nanoparticles concentration $\varphi (\eta )$ is a rising function of the thermophoresis parameter and a decreasing function of the Brownian movement parameter. The increase in the thermophoresis factor $Nt$ provides a logic and physical interpretation to the reduction in $\varphi$, where the nanoparticles scatter and accelerate in their accidental movement with the rise of $Nt$ as displayed in Fig. 26. Furthermore, the Brownian motion represents a measure of the random motion of the nanoparticles scattered in a fluid. This random movement rises with the rise of $Nb$, which represents more departure of the nanoparticles as obtained in Fig. 27. Moreover, the Brownian motion inclines to move nanoparticles from areas of high concentration to areas of low concentration. This result agrees with the findings of Ramesh et al.^53^, Abou-zeid^12^, Abou-zeid and Mohamed^57^, Alebraheem and Ramzan^58^.

### Skin friction, Nusselt and Sherwood parameters

Table 1 is designed to discuss the influences of the factors $n$, $M$ and $We$ on the skin friction coefficient $Cf_{y}$ and compare its values with the previous concluding data of Zakir Ullah et al.^36^, and Akbar et al.^45^ to confirm the correctness of the current numerical structure. As obtained in Table 1, there is a good agreement with the works of Zakir Ullalh et al.^36^ and Akbar et al.^45^. It is shown that the skin friction decreases with the rise of $n$, whereas it grows with the increase of $M$ and is not affected by the change of $We$. Moreover, Table 2 illustrates the Nusselt numeral in the case of $\lambda = \alpha = S = 0$ for various values of $M$, $n$ and $We$. Table 3 clarifies the Sherwood numeral in the case of $b_{1} = b_{2} = 0,$ and $N_{b} = 1$ for various values of $R_{2}$, $N_{t}$ and $Le$. It is found that the Nusselt numeral decays with the parameters $M$, $n$ and $We$, as seen in Table 2. Furthermore, the Sherwood numeral decreases with $R_{2}$, $N_{t}$ and increases with $Le$.

**Table 1:** Skin friction indices are compared to the body of available research when $\lambda = \alpha = S = 0$ for various values of $M$, $n$ and $We$.

Skin friction coefficient										
$n$↓	$M$↓	Zakir Ullalh et al.^36^			Akbar et al.^45^			Present results		
		$We$ = 0.0	$We$ = 0.3	$We$ = 0.5	$We$ = 0.0	$We$ = 0.3	$We$ = 0.5	$We$ = 0.0	$We$ = 0.3	$We$ = 0.5
0.0	0.0	1	1	1	1	1	1	1.0004	1.0004	1.0004
0.1	0.0	0.94868	0.94248	0.93826	0.94868	0.94248	0.93826	0.94901	0.9428	0.93858
0.2	0.0	0.89442	0.88023	0.87026	0.89442	0.88023	0.87026	0.89464	0.8804	0.87046
0.3	0.5	1.02472	0.98804	0.96001	1.09544	0.98804	0.96001	0.93543	0.9047	0.88150
0.3	1.0	1.18322	1.13454	1.09616	1.26491	1.13454	1.09616	1.18322	1.1345	1.09616

**Table 2:** Nusselt numeral for $\lambda = \alpha = S = 0$ with various values of $M$, $n$ and $We$.

*n*↓	*We*↓	$- \theta^{\prime } (0)$		
		*M* = 0.0	*M* = 0.3	*M* = 0.5
0.0	0.0	$0.31948$	$0.31392$	$0.30503$
0.1	0.0	$0.31261$	$0.30704$	$0.29818$
0.2	0.0	$0.30493$	$0.29939$	$0.29061$
0.3	0.5	$0.29061$	$0.2848$1	$0.27568$
0.3	1.0	$0.28318$	$0.27675$	$0.26648$

**Table 3:** Sherwood numeral for $b_{1} = b_{2} = 0,N_{b} = 1$ with various values of $R_{2}$, $N_{t}$ and $Le$.

$R_{2}$↓	*N*_*t*_↓	$- \varphi^{\prime } (0)- \varphi^{\prime } (0)$		
		$Le$ = 0.1 $Le$ = 0.5 $Le$ = 0.7		
0.0	0.0	$0.20392$	$0.37315$	$0.45895$
0.1	0.0	$0.20387$	$0.37293$	$0.45865$
0.2	0.0	$0.20383$	$0.3727$ 5	$0.45839$
0.3	0.5	$0.20352$	$0.37112$	$0.45612$
0.3	1.0	$0.20294$	$0.36807$	$0.45187$

## Concluding remarks

In accordance with the numerous applications of stretching sheets in manufacturing and production processes, the present study is prepared to introduce valuable results in this field of research. The work is concerned with the numerical analysis of an incompressible tangent-hyperbolic micropolar nanofluid movement past a stretching horizontal layer throughout a permeable medium. The novelty of the current work comes from the impact of a normal unvarying magnetic strength, Ohmic dissipation, temperature generation, and chemical reaction with the prescribed prototype of nanofluid flow. To reduce the mathematical analysis of the model, a convenient similarity transform is utilized to convert the partial differential equations to ordinary ones. Several non-dimensional physical numbers are explored, which play important roles and control the targeting distributions. Subsequently, a set of figures and numerical tables has been analyzed to demonstrate the implication of the various relevant physical parameters. The numerical analysis is performed in light of the shooting technique with the aid of Mathematica program version 11 to construct predictable distributions of all typical significant functions concerning velocity, microrotation (angular) speed, heat, and nanoparticles concentration. The foremost findings of the current work may be summarized in the following points:

- The impacts of the different factors on both the radial and angular velocities are similar. It is found that $M$, $We$ and $n$ decrease them, whereas $K$, $\lambda$ and $Da$ increase them.
- The temperature transmission rises with the raise of the parameters $M$, $n$,$\,N_{T}$, $\,N_{B} \,$, $Ec$ and $R$. On the other hand, the growth of the parameters $\Pr$ and $\lambda$ plays a dual role in heat transfer.
- The nanoparticles distribution $\varphi$ rises with the rise in the values of $M$, $Nt$ and $\alpha$, meanwhile it declines with the rise of $Nb$ and $R_{2}$. Like heat transfer, the parameter $\lambda$ plays a dual role in $\varphi$, but an opposite one.
- Some quantitative values of the skin friction factor for different values of $M$, $n$, and $We$ are concluded and compared with some previous studies.
- Some measureable values of Nusselt and Sherwood numbers are tabulated for different parametric values.

## Data Availability

All data generated or analyzed during this study are included in this manuscript.

## List of symbols

$n$: Power law index
$$u_{i}$$: Velocity components (m . s^−^^1^)
$$x_{j}$$: Model coordinates (m)
$f_{i}$: Body force (N = kg . m . s^−2^)
$N$: Microrotation vector (kg . m^2^/s)
$k\,$: Vortex viscosity coefficient
$T$: Temperature of fluid
$$D_{B}$$: Brownian diffusivity
$C$: Nanoparticles concentration (Mol . m^−3^)
$$D_{T}$$: Thermophoretic diffusion (m^2^ . s^−^^1^)
$T_{\infty }$: Temperature at infinity (K(Kelvin))
$q_{r}$: Radiative heat flux
$J_{i}$: Electric current density components
$Q_{0}$: Heat source/sink parameter
$R_{1}$: Chemical reaction parameter
$$L$$: Permeability parameter (H/m)
$T_{w}$: Temperature at wall (K(Kelvin))
$C_{w}$: Nanoparticles concentration at wall (Mol . m^−3^)
$C_{\infty }$: Nanoparticles concentration at infinity (Mol . m^−3^)
$$\Pr$$: Prandtl numeral
$${\text{Le}}$$: Lewis’s numeral
$$N_{T}$$: Thermophoresis factor
${\text{Nb}}$: Brownian motion factor
$${\text{We}}$$: Weissenberg parameter
$Q$: Heat source parameter (m^3^ . s^−1^)
$${\text{Ec}}$$: Eckert numeral
$R$: Thermal radiation factor
$${\text{Da}}$$: Darcy numeral
$b_{1}$: Thermal slip parameter
$b_{2}$: Nanoparticles slip parameter
$R{}_{1},R_{2}$: Chemical reaction parameters (Mol/(m^3^ . s))

## Greek symbols

$\overline{\tau }$: Tensor of extra stress
$\mu_{\infty }$: Endless shear rate viscosity
$\mu_{0}$: Zero-shear rate viscosity
$\Gamma$: Time-dependent material continual
$\rho$: Fluid density (Kg/m^3^)
$$\gamma$$: Microrotation parameter
$(\rho c)_{f}$: Heat capacity of the flow (J . K^−1^)
$(\rho c)_{p}$: Heat capability of the nanoparticles (J . K^−1^)
$\alpha$: Coefficient of thermal diffusivity
$$\sigma$$: Electrical conductivity (S/m)
$$\nu$$: Kinematic viscosity (m^2^/s)
$\beta_{1}$: Coefficient of heat slip
$\beta_{2}$: Coefficient of mass slip
$$\lambda$$: Stretching parameter
$$\alpha$$: Velocity slip
